# Targeting Complement C3a Receptor to Improve Outcome After Ischemic Brain Injury

**DOI:** 10.1007/s11064-021-03419-6

**Published:** 2021-08-11

**Authors:** Marcela Pekna, Anna Stokowska, Milos Pekny

**Affiliations:** 1 Laboratory of Regenerative Neuroimmunology, Center for Brain Repair, Department of Clinical Neuroscience, Institute of Neuroscience and Physiology, Sahlgrenska Academy, University of Gothenburg, Box 440, 405 30 Gothenburg, Sweden; 2 Laboratory of Astrocyte Biology and CNS Regeneration, Center for Brain Repair, Department of Clinical Neuroscience, Institute of Neuroscience and Physiology, Sahlgrenska Academy, University of Gothenburg, Gothenburg, Sweden

**Keywords:** C3a, C3a receptor, The complement system, Brain ischemia, Ischemic stroke, Hypoxic-ischemic encephalopathy, Birth asphyxia, Neural plasticity, Neuroprotection, Recovery

## Abstract

Ischemic stroke is a major cause of disability. No efficient therapy is currently available, except for the removal of the occluding blood clot during the first hours after symptom onset. Loss of function after stroke is due to cell death in the infarcted tissue, cell dysfunction in the peri-infarct region, as well as dysfunction and neurodegeneration in remote brain areas. Plasticity responses in spared brain regions are a major contributor to functional recovery, while secondary neurodegeneration in remote regions is associated with depression and impedes the long-term outcome after stroke. Hypoxic-ischemic encephalopathy due to birth asphyxia is the leading cause of neurological disability resulting from birth complications. Despite major progress in neonatal care, approximately 50% of survivors develop complications such as mental retardation, cerebral palsy or epilepsy. The C3a receptor (C3aR) is expressed by many cell types including neurons and glia. While there is a body of evidence for its deleterious effects in the acute phase after ischemic injury to the adult brain, C3aR signaling contributes to better outcome in the post-acute and chronic phase after ischemic stroke in adults and in the ischemic immature brain. Here we discuss recent insights into the novel roles of C3aR signaling in the ischemic brain with focus on the therapeutic opportunities of modulating C3aR activity to improve the outcome after ischemic stroke and birth asphyxia.

## Introduction

Each year, stroke affects about 15 million people worldwide. 50% of the approximately 10 million stroke survivors suffer from long-lasting or permanent functional impairment, which makes stroke the primary cause of disability in adults. Stroke most commonly results from the occlusion of a major vessel in the brain. If the occlusion is not rapidly reversed, an infarct develops due to the death of all cells in the affected tissue. Only a small fraction of patients arrive at the hospital in time to be eligible for blood clot removing procedures. Rehabilitation - that only rarely leads to full recovery - remains as the only option for the majority of stroke survivors. Therefore, improving recovery of function by effective neuroprotection, and plasticity- and regeneration-promoting strategies has become a major research focus.

Loss of function after stroke is due to cell death in the infarcted tissue and cell dysfunction in the surrounding as well as remote brain regions that are connected to the damaged area. Recovery of function involves reversal of dysfunction, activation of cell repair (cell genesis, axonal regeneration), functional reorganization within existing networks (changing the properties of existing neural pathways) and neuroanatomical plasticity leading to the formation of new connections (axonal sprouting, synaptogenesis). Some of these mechanisms, jointly called neural plasticity, are involved in normal learning, are enhanced by the milieu created following the injury, and contribute to recovery of function after stroke and other CNS injuries [1].

Hypoxic–ischemic encephalopathy is one of the most critical pathologic conditions in neonatal medicine. Neonatal hypoxic–ischemic encephalopathy due to perinatal asphyxia is the leading cause of neurological disability resulting from birth complications. It is caused by the disruption of blood flow and oxygen supply to the brain prior to or during delivery and occurs in 1–2 of 1000 live term births. Recent advances in critical care have improved the survival of infants suffering from hypoxic–ischemic encephalopathy, but approximately 50% of survivors will develop complications such as mental retardation and cerebral palsy. Long-term neurological impairment after neonatal hypoxia-ischemia correlates with the extent of brain damage [2]. Perinatal asphyxia and other perinatal brain insults lead to neuronal cell death in the acute and secondary phases, which last for hours to days; delayed neuronal cell death in the so-called tertiary brain damage phase, which can persist for weeks to years, prevents repair and regeneration, disturbs the development and function of affected brain networks, or sensitizes them to dysfunction and cell death due to a subsequent inflammatory challenge [3]. Even a mild-to-moderate ischemic insult can result in progressive cerebral atrophy, delayed infarction, and long-term cognitive impairment in rodent hypoxic–ischemic encephalopathy models [2, 4–8]. However, the underlying mechanisms are not fully understood. Therapeutic hypothermia is a clinically accepted therapy for hypoxic–ischemic encephalopathy, however, treatment of eight children is required for one child to be saved from the development of severe disability. Therapies to further improve outcomes of infants suffering from acute encephalopathy are therefore urgently needed [9].

The complement system is an important constituent of the humoral innate immune response best known for its role in the elimination of pathogenic bacteria and initiation of inflammation. The complement system consists of more than 50 soluble proteins, cell receptors and control proteins found in the blood and tissues. Their specific roles in innate immunity include the opsonization and lysis of pathogens, elimination of soluble antigen–antibody complexes, removal of dead cells and tissue debris, stimulation of leukocyte chemotaxis, and initiation of inflammation. Through the regulation of B and T lymphocyte functions, complement affects also adaptive immunity [10]. Hepatocytes are the main source of soluble complement proteins, however many complement factors and receptors are also expressed locally in the brain and spinal cord [10]. The complement system mediates the reciprocal signaling between the cells in the CNS and acts as both the modulator and effector of their functions [11].

C3a receptor (C3aR) is a G-protein-coupled receptor for a cognate complement-derived peptide C3a [12]. C3aR is expressed in many tissues including the brain [13, 14]. Besides its many functions in the regulation of inflammation [15], C3aR has been shown to play a role in the development and normal function of the CNS, however excessive C3aR signaling has been implicated as a factor in neurodegeneration. In the ischemic brain, signaling through C3aR can both contribute to tissue damage and stimulate neural plasticity responses involved in functional recovery. C3aR represents an attractive target for the treatment of ischemic brain injury, however, for the optimal outcome neurodevelopmental stage, the mode of interaction as well as timing of the intervention seem to be of critical importance.

## C3aR in CNS Development and Function

**Fig. 1:**
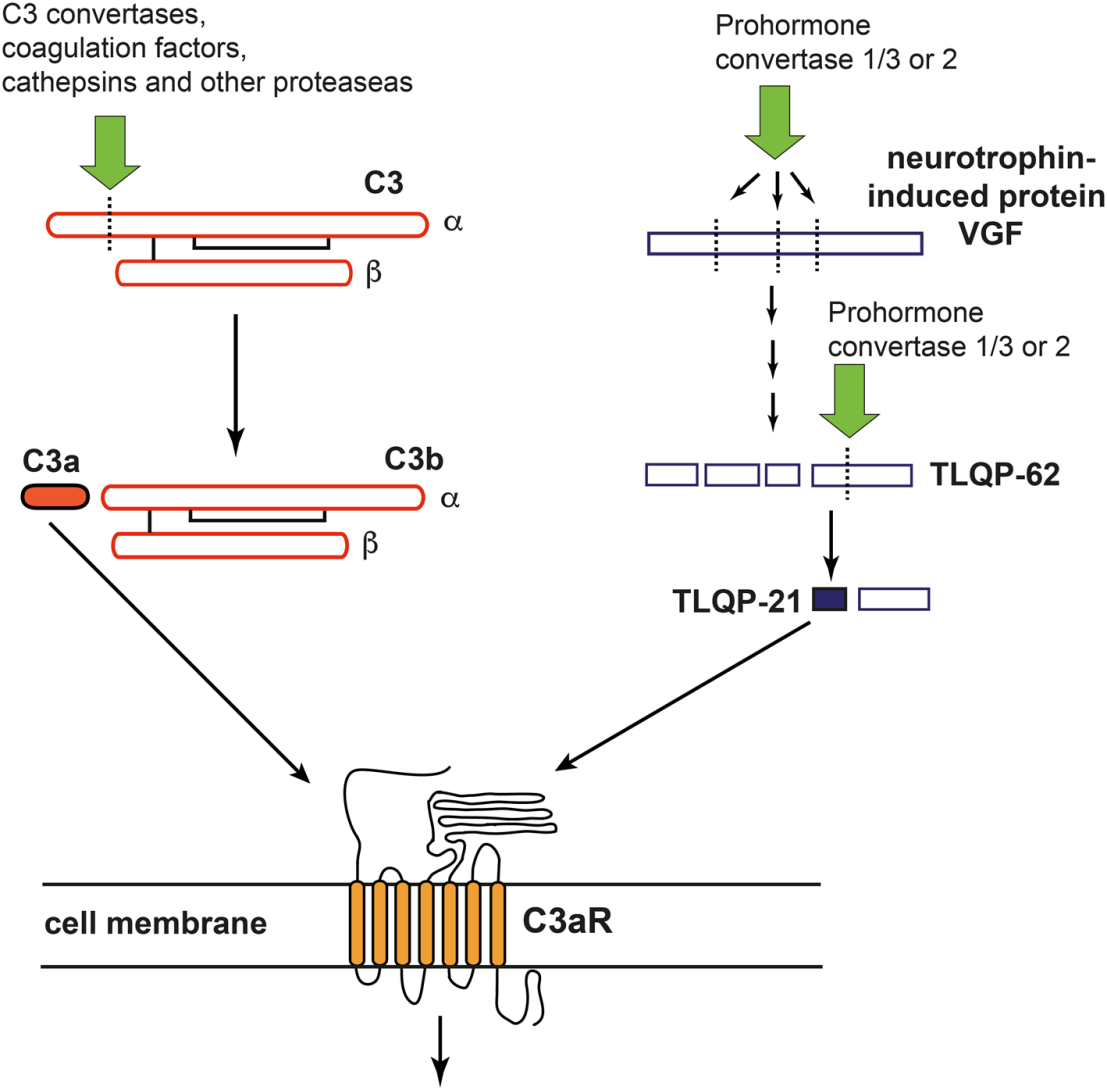
C3a and TLQP-21 are endogenous ligands of C3aR in the CNS. C3a can be released through the proteolytic activation of C3 by C3-convertases, coagulation factors XIa, Xa, IXa, thrombin, and plasmin, cathepsins and a number of other membrane-associated or serine proteases such as mannan-binding lectin-associated serine protease 1, neutrophil elastase, granulocyte neutral proteases, lysosomal enzymes, and kallikrein. The neuropeptide TLQP-21 is derived from the neurotrophin-inducible protein VGF through a stepwise proteolytic cleavage by prohormone convertases 1/3 and 2

C3aR is a member of the rhodopsin family of seven transmembrane G-protein-coupled receptors [12]. As the name implies, C3aR was identified as the receptor for C3a, a 9 kDa, 77 amino acid peptide and the smaller of the two activation fragments generated through the proteolytic activation of the third complement component (C3), the central molecule of the complement system [12]. Beside the so called C3-convertases, i.e. enzymatic complexes generated by the complement cascade triggered by e.g. danger associated signals on the surface of pathogenic microorganisms, C-reactive protein and amyloid-β [16], C3a can be released through the proteolytic activation of C3 by a number of other membrane-associated or serine proteases such as mannan-binding lectin-associated serine protease 1 [17], neutrophil elastase, cathepsins [18, 19], granulocyte neutral proteases [20], lysosomal enzymes, kallikrein, as well as coagulation factors XIa, Xa, IXa, thrombin, and plasmin [21, 22]. In addition to C3a, C3aR has been shown to bind the neuropeptide TLQP-21 [23], that is derived from the neurotrophin-inducible protein VGF through proteolytic cleavage by prohormone convertases 1/3 and 2 [24], Fig. 1.

C3aR is expressed by embryonic stem cells [25], neural progenitor cells [26] and mature neurons [27–30]. In neural progenitor cells, C3a-C3aR signaling activates the extracellular signal-regulated kinase (ERK)1/2 signaling pathway, regulates neuronal differentiation, neuronal maturation and migration [31]. C3a was also shown to accelerate the migration of granule cells of the developing cerebellum [32] and regulate neuronal migration during cortical development [33]. The neurodevelopmental role of C3aR signaling is evidenced by altered brain morphology, cognitive defects and hyperactive behavior observed in adult mice constitutively lacking C3aR [25, 34]. C3aR stimulates neurogenesis in adult naïve mice [26] and normal level of neuronal C3aR signaling is required for synaptic plasticity and maintenance of normal dendritic extensions [35].

Astrocytes express C3aR [27, 28, 36] and respond to C3a by activation of intracellular signaling [37] and the expression of cytokines such as interleukin (IL)-6, IL-8 and nerve growth factor (NGF) [38–40].

Microglia express C3aR and C3a stimulation of microglia triggers an increase in intracellular calcium concentration [41] and upregulation of NGF [42]. C3aR-mediated signaling regulates the phagocytic activity of microglia, short exposure to C3a stimulates and chronic C3a treatment reduces microglial phagocytosis [43]. Activation of microglial C3aR by TLQP-21 in the dorsal horn of the spinal cord has been implicated in spinal neuroplasticity and neuropathic pain [44], however the functions of TLQP-21 in the brain and its role in the brain responses to ischemia have not been studied.

C3aR signaling increases vascular permeability, stimulates smooth muscle contraction, and triggers the activation and directed migration of inflammatory cells [13]. C3aR regulates endothelial cell expression of cytokines and adhesion molecules, which are important for leukocyte recruitment into the brain, and control blood brain barrier permeability [45–47]. Activation of C3aR signaling on epithelial cells of the choroid plexus can lead to the disruption of blood-cerebrospinal fluid barrier [48].

The multiple effects of C3aR signaling on the function of the different cell types in the CNS are summarized in Table 1.

**Table 1:** The effects of C3aR signaling on the cells in the CNS

Cell type	C3aR functions	References
Neural stem / progenitor cells	Neuronal differentiation Migration	[31] [31]
Neurons	Migration Neurite outgrowth Modulation of synaptic strength Modulation of dendritic morphology	[32, 33] [31] [35] [35]
Astrocytes	Cytokine expression Survival	[38–40] [36]
Microglia	NGF upregulation Regulation of phagocytosis	[42] [43]
Endothelial cells	Cytokine expression Expression of cell adhesion molecules	[45] [46]
Epithelial cells of the choroid plexus	Disorganization of tight junctions	[48]

## C3aR and Intracellular Signaling

**Fig. 2:**
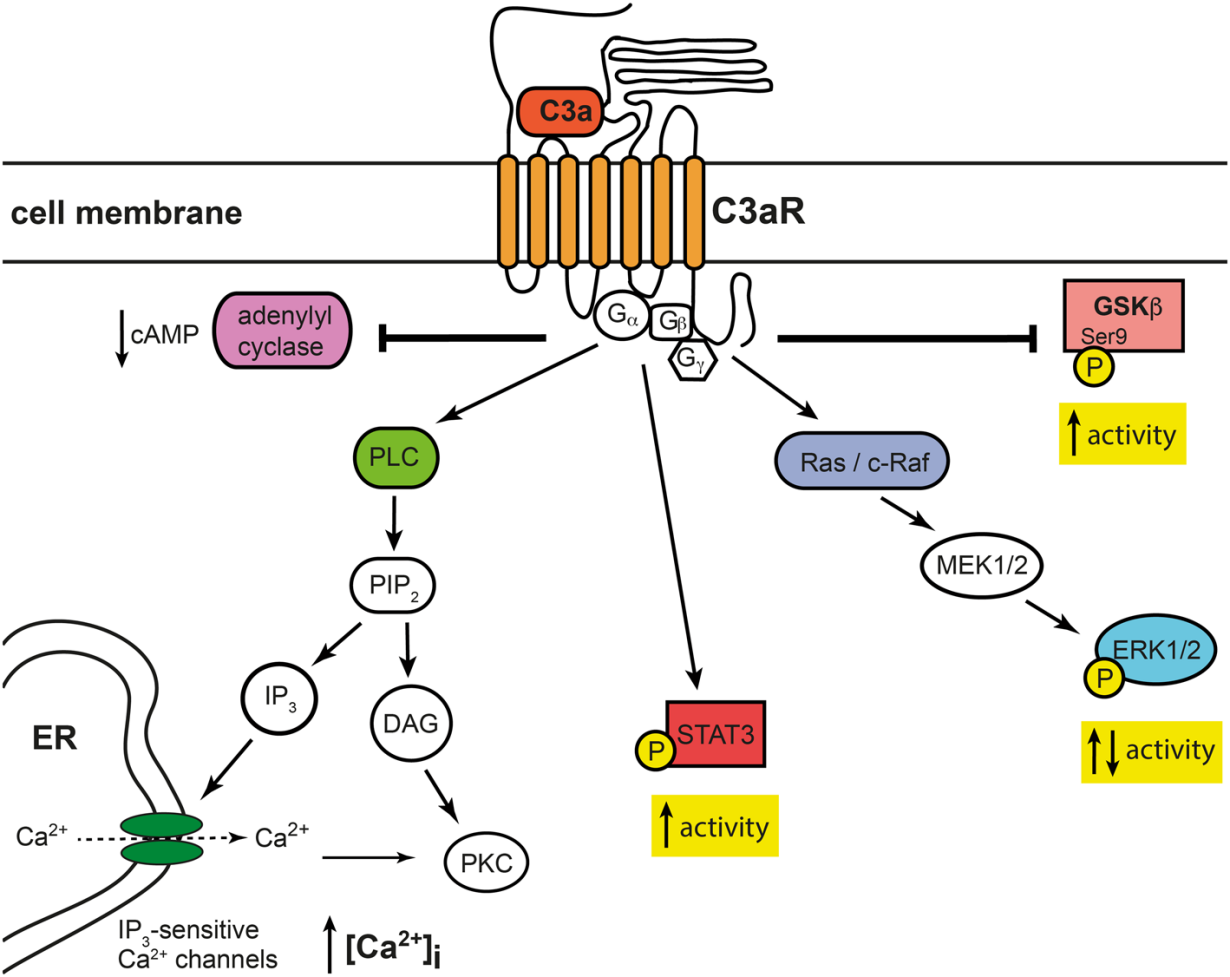
Intracellular signaling pathways regulated by C3aR in the cells of the CNS. In microglia, astrocytes and endothelial cells, C3aR signaling inhibits the adenylyl cyclase pathway and reduces the inhibitory Ser9 phosphorylation of glycogen synthase 3β (GSK3β). C3aR signaling activates the phospholipase C pathway, modulates the activity of the extracellular signal regulated kinase 1/2 (ERK1/2) pathway, and activates STAT3

In various cell types such as microglia, astrocytes and endothelial cells, C3aR activates the phospholipase C pathway leading to the opening of intracellular calcium channels and increase in the intracellular calcium levels [37, 41, 47]. C3a-C3aR signaling modulates the activity of the ERK1/2 pathway including Ras and c-Raf [31, 37, 49]. At least in astrocytes, stimulation of C3aR leads to the inhibition of the adenylyl cyclase pathway [37]. In microglia, C3a-C3aR signaling was shown to increase the phosphorylation and activation of STAT3 [50]. C3aR antagonist SB290157 reduced the inhibitory Ser9 phosphorylation of glycogen synthase 3β (GSK3β) in the SH-SY5Y neuroblastoma cells [51], pointing to the role of C3aR signaling in the regulation of GSK3β activity, Fig. 2.

## The Roles of C3aR in the Ischemic Brain

Astrocytes, microglia and neurons are the source of complement proteins in the CNS [52–55]. After brain ischemia, pronounced complement activation was reported both in the systemic circulation of human patients [56–60] and in the human post-mortem brain tissue [61, 62]. Experimental studies implicated C3a as the key mediator of brain tissue injury in the acute phase after focal brain ischemia [63]. However, given the broad expression of C3aR on the different cell types in the CNS, and the potential involvement of C3aR in the regulation of several intracellular signaling pathways, the net effect of C3aR activation on the long-term outcome may depend on the specific cell type and the timing of the response in relation to the ischemia onset. For example, the expression of C3aR by astrocytes is increased by ischemia [27, 28, 36], and C3a was shown to promote astrocyte survival after ischemia through its inhibitory effect on ERK signaling-mediated apoptotic pathway and caspase-3 cleavage [36]. C3a protects neurons against excitotoxicity-induced cell death, but only when neurons are co-cultured with astrocytes [64]. Further, microglial cells treated with C3a exhibit neuroprotective phenotype as evidenced by increased production of NGF [42].

### The Effects of C3aR Signaling in the Acute Phase After Ischemic Brain Injury

In the first days after stroke, C3a levels in blood are elevated and in some stroke subtypes show association with unfavorable outcome [57, 59, 60, 65]. The involvement of C3 in the pathophysiology of ischemic stroke is also supported by human genetic studies [66]. Indeed, C3a-C3aR signaling was shown to regulate bleeding time after tail injury and thrombosis in mice, and C3aR deficient mice were less prone to experimental stroke and myocardial infarction [67]. In the acute phase after stroke, endothelial activation and leukocyte recruitment into the brain are reduced in mice lacking C3 and C3aR [46]. C3 deficiency and pre-treatment of mice with C3aR antagonist reduced granulocyte infiltration, infarct volume and neurological deficit scores assessed 24 h after transient cerebral ischemia [63], and mice that were treated with SB290157, a C3aR antagonist [68], starting before the induction of transient ischemia developed smaller infarcts as assessed 7 days after ischemia [69]. Another study demonstrated that the C3aR antagonist pre-treatment reduced the expression of ICAM-1 protein on endothelial cells and granulocyte infiltration to the brain parenchyma [70]. Even when administered 2 h after the induction of cerebral ischemia, C3aR antagonist treatment reduced functional impairment, infarct volume, edema and hemorrhagic transformation assessed 48 h later [71]. In an in vitro ischemia model, C3a led to increased endothelial permeability [72], and C3aR antagonist administration preserved the integrity of endothelial cell tight junctions and reduced the activation of ERK, suggesting that endothelial C3aR may act via ERK signaling [73]. These results point to therapeutic benefits of systemic inhibition of C3aR signaling in the acute phase after ischemic injury to the adult brain through mitigating the pro-inflammatory effects of C3a-C3aR signaling on endothelial cells and reducing the recruitment of inflammatory cells from the systemic circulation.

In contrast to the ischemic injury to the adult brain, in a model of neonatal hypoxic-ischemic brain injury, mice expressing biologically active C3a under the control of the glial fibrillary acidic protein promoter *(GFAP*-*C3a), i.e.* expressing C3a in reactive astrocytes, showed reduced brain tissue loss assessed 3 weeks later [7]. In the same study, single intraventricular injection of C3a mitigated cognitive function impairment due to neonatal hypoxia-ischemia in control mice but not in mice lacking C3aR (*C3aR*^*−/−*^*)* 6 weeks later [7]. Importantly, mice that received intranasal treatment with C3a once daily for 3 days starting 1 h after hypoxia-ischemia induction at postnatal day 9 were protected against cognitive impairment observed in vehicle treated mice 6 weeks after hypoxia-ischemia [8]. Thus, in the acutely injured immature brain, C3a-C3aR signaling appears to promote recovery. Therapeutic hypothermia, the only intervention that improves clinical outcome after neonatal hypoxic-ischemic encephalopathy, was shown to increase the levels of C3a in the brain and plasma, and to lead to the upregulation of C3aR in the brain in a rat model of hypoxic-ischemic encephalopathy [74]. These results point to C3a-C3aR signaling as a mediator of the neuroprotective effects of hypothermia. Together, these studies support the notion that the cellular functions of C3aR signaling differ profoundly depending on the developmental stage of the neural tissue.

### C3a-C3aR and Post-stroke Neural Plasticity

Ischemic injury to the brain is known to trigger a range of endogenous plasticity and repair processes, including proliferation, differentiation and migration of neural stem and progenitor cells [75–77], axonal sprouting, dendritic arborization and synaptogenesis, that lead to rewiring of the existing neuronal connections and the formation of new ones [78, 79]. The structural and functional constituents of ischemia-induced neural plasticity are recognized as critically important contributors to recovery of function after stroke and other CNS injuries [1]. There is growing evidence for the role of C3a-C3aR signaling in stimulating adaptive neural plasticity responses after ischemic brain injury. These findings point to the use of C3aR agonists as a therapeutic strategy to facilitate functional recovery in the post-acute and chronic phase after ischemic brain injury, Table 2.

**Table 2:** The functions of C3a in the acute, post-acute and chronic phase after ischemic brain injury

	Function	References
Acute phase	Leukocyte recruitment	[46, 63, 69, 70]
	Inflammatory endothelial activation	[46, 70]
	Endothelial cell and blood-brain barrier dysfunction	[71–73]
Post-acute and chronic phase	Post-stroke neurogenesis	[26, 80]
	Post-stroke synaptogenesis	[89]
	Post-stroke expression of GAP-43, marker of axonal and glial plasticity	[89]
	Modulation of reactive gliosis	[8]
	Neuroprotection, survival of astrocytes after ischemic stress	[7, 36]

#### C3aR and Post-stroke Neurogenesis

C3aR is expressed by hippocampal neural stem cells in vitro as well as migrating neuroblasts in vivo [26], and in vitro studies show that C3a stimulates neural progenitor cell differentiation [31]. C3a also regulates the migration of adult neural progenitor cells in response to other environmental clues such as stromal derived factor 1α [31]. The contention that C3aR signaling acts as a positive regulator of adult neurogenesis is further supported by in vivo evidence showing that hippocampal and subventricular zone neurogenesis is impaired in mice constitutively lacking C3aR or C3, and mice treated with C3aR antagonist SB 290157 [26]. The stimulatory effect of C3aR signaling on basal adult neurogenesis was confirmed by other investigators, who observed reduced number of proliferating doublecortin-positive neural progenitor cells in the subventricular zone of unchallenged mice treated with the same C3aR antagonist [69].

Despite larger infarct, the C3 deficient mice had reduced neurogenic response in the ipsilesional subventricular zone and in the peri-infarct region after focal cerebral ischemia induced by middle cerebral artery occlusion at both 7 and 21 days after ischemia [26]. These findings point to C3 activation products as positive regulators of post-stroke neurogenesis and neuroprotection. In a model of ischemic stroke induced by photothrombosis - which results in ischemic lesion with minimal or no penumbra, and thus allows to study the effects of C3aR signaling on neural plasticity and functional recovery independent of neuroprotection - we showed that C3a overexpression in the *GFAP*-*C3a* mice increased whereas C3aR deficiency decreased the number of newly born neurons in the peri-infarct region on day 21 after stroke despite comparable infarct volumes [80]. These results strongly support the contention that C3a-C3aR signaling stimulates the stroke-induced neurogenic response. Although the activity of the *GFAP* promoter and thus of the C3a transgene is too low to affect the levels of basal hippocampal and subventricular zone neurogenesis in unchallenged adult *GFAP*-*C3a* mice [81], pronounced and persistent reactive gliosis in the peri-infarct tissue [82] results in sufficiently high transgene-derived C3a levels to impact post-stroke neurogenesis in this region.

On the other hand, daily systemic treatment with a low dose of the C3aR antagonist SB 290157 starting before the induction of transient focal cerebral ischemia increased the proliferation of neuronal precursor cells in the ipsilesional subventricular zone 7 days later [69]. As argued by the authors, in the absence of any effect of the same treatment protocol on subventricular zone neurogenesis in unchallenged mice, the positive effect of low dose C3aR antagonist treatment on post-stroke neurogenesis is conceivably due to the inhibition of the inflammation including the reduced recruitment of activated T-lymphocytes rather than to the direct effect of the drug on the progenitor cells [69]. The specific mechanism of C3a generation in an unchallenged neurogenic niche and the mechanisms of C3a generation in the post-acute and chronic phase after stroke need to be elucidated in future studies.

#### C3aR and Post-stroke Synaptic Plasticity

In the developing brain, the complement system is involved in the regulation of the number of neuronal synapses. Specifically, transforming growth factor β that is secreted by immature astrocytes triggers the neuronal expression of complement component C1q in the developing visual thalamus [83, 84] and sensorimotor cortex [85]. Binding of C1q to externalized phosphatidylserine [86] leads to the deposition of C3b which tags the synapse for recognition by microglial complement receptor 3 (CR3) and subsequent elimination by phagocytosis [83, 87]. However, given that the development and experience-dependent plasticity of the binocular zone of the primary visual cortex was not altered in mice lacking C1q, the contribution of C1q to synapse elimination is not universal and instead appears to be context-dependent [88].

While the C3b fragment of C3, through its interaction with the CR3, drives the removal of neuronal synapses, at least during the CNS development, neuronal C3aR signaling promotes increase in synaptic strength through membrane localization of the α-amino-3-hydroxy-5-methyl-4-isoxazolepropionic acid receptor [35]. In addition, dendritic complexity is reduced in mice lacking neuronal C3aR as well as in mice treated with a C3aR antagonist [35]. On the other hand, excessive activation of neuronal C3aR can lead to reduced complexity of dendrites and impair synaptic function [35]. In the post-stroke brain, C3a overexpression in the *GFAP*-*C3a* mice increased whereas C3aR deficiency decreased the density and size of glutamatergic pre-synaptic terminals in the peri-infarct region as well as in the contralesional hemisphere in a manner that was cortical region- and cortical layer-specific. None of these parameters was altered in an unchallenged brain of these genetically modified mice [89]. The synaptogenic function of C3a-C3aR signaling in the post-stroke adult brain was further supported by the finding that mice that received daily intranasal treatment with C3a for 14 to 21 days starting 7 days after ischemia induction had higher density of pre-synaptic terminals and faster functional recovery that was sustained 4 weeks after cessation of the treatment [89].

#### C3aR and Post-stroke Axonal Plasticity

As a hallmark of CNS regeneration, axonal sprouting and plasticity are associated with reactivation of the intrinsic neuronal growth program and upregulation of the membrane phosphoprotein growth associated protein (GAP)-43 [90]. GAP-43 associates with axonal growth cones, is upregulated during reactive synaptogenesis and is used as marker of axonal sprouting and plasticity [91–93]. Astrocyte-derived GAP-43 has been shown to promote neuronal survival and glial plasticity [94]. The stroke-induced increase in GAP-43 expression in the peri-infarct region was reduced in mice constitutively lacking C3aR, while it was further increased when C3a was expressed in reactive astrocytes or administered intranasally starting 7 days after stroke [89]. In light of the ischemia-induced upregulation of C3 in sprouting neurons [95] and the stimulatory effect of C3a on neurite outgrowth in vitro [31], these findings implicate C3a-C3aR as a contributing factor in post-stroke axonal plasticity.

### C3aR and Glial Responses to Ischemia

Given the evidence for the involvement of neuronal C3aR in the modulation of synaptic strength and dendritic morphology [35], and the role of C3a-C3aR in neural progenitor cell differentiation and migration [31], the increase in peri-infarct neurogenesis, upregulation of expression of GAP-43 and increased number of pre-synaptic terminals, particularly glutamatergic terminals in C3a overexpressing and C3a treated mice [80, 89], are arguably at least in part due to a direct effect of C3a on neurons. However, given its broad expression and the multitude of functions of C3aR in the different cell types in the brain (Table 1), the effects of C3a-C3aR signaling on the plasticity of the post-ischemic brain can be also indirect through the modulation of the functions of astrocytes, microglia, endothelial cells, stem / progenitor cells, and the epithelial cells in the choroid plexus.

Within the first hour after the ischemia onset, microglia become activated [96] and the density of activated microglia/macrophages in the periphery of the ischemic lesion is increased for several weeks [97]. Astrocytes in the peri-infarct region change their expression profile [98], proliferate and form a glial scar that restricts the damaged area and prevents the infiltrating leukocytes from spreading into the surrounding healthy parenchyma [99–103]. Peri-infarct reactive gliosis persists for at least several weeks [82]. Genetic attenuation of reactive gliosis achieved by ablation of genes coding for intermediate filament (nanofilament) proteins GFAP and vimentin [104–106], markers of astrocyte reactivity [103, 107], led to more pronounced neuronal loss in the acute phase after ischemic stroke [99] and after retinal ischemia-reperfusion [108], but not in neonatal hypoxic-ischemic brain injury [109]. Reduced expression of GFAP in the peri-infarct cortex was associated with increased axonal sprouting and better functional recovery 7 days after ischemic stroke [110]. In the post-ischemic brain, astrocytes participate in several aspects of remodeling of the neural tissue and the peri-infarct networks, such as phagocytic clearance of tissue debris, formation of new synapses and neurogenesis [111–114]. Our findings that intranasal treatment with C3a improved functional outcome and reduced the expression of GFAP and the density of microglia/macrophages in the ipsilesional hippocampus after neonatal hypoxic-ischemic brain injury [8], suggest that the protective effects of C3a-C3aR signaling in the immature brain may be mediated through the modulation of glial responses. The functions of C3a-C3aR signaling in the regulation of reactive gliosis in the context of ischemic brain injury in adults and a detailed characterization of the impact of C3a-C3aR signaling on the phenotypes of reactive astrocytes [115] remain to be investigated.

The multiple roles of C3a-C3aR signaling in CNS responses to ischemic injury are summarized in Table 2.

## C3aR and Neurodegeneration

The complement system is an important driver of age-related synapse loss and cognitive decline [116], and a prominent factor in neurodegeneration [35, 50, 117–121]. The elimination of synapses mediated by C3b-CR3 may be re-activated in neurodegenerative diseases such as glaucoma [122] and Alzheimer’s disease [118]. C3 deficient mice have better hippocampus-dependent learning and memory functions [123], and are protected from age-related region-specific loss of neurons and synapses in the hippocampus, age-related cognitive decline [116], and axotomy-induced inhibitory synapse removal [124]. In spite of higher amyloid-β plaque load, C3 deficient mice were also protected against Alzheimer type of neurodegeneration and cognitive decline [125]. These results implicate the involvement of the complement system and in particular C3, in amyloid-β clearance and amyloid-β induced synapse elimination. Identical or similar mechanisms can operate also in the post-stroke brain. Indeed, through a number of mechanisms, including impaired perivascular space integrity, reduced efficiency of the glymphatic system, inflammation, hypoxia, and blood-brain barrier dysfunction, stroke can accelerate amyloid-β deposition in brain parenchyma, which in turn leads to synaptic dysfunction, cognitive decline and dementia (reviewed in [126]).

Neuronal death, reactive gliosis, and axonal degeneration occur also after stroke in remote brain regions that were not directly affected by the ischemic injury but had synaptic connections with neurons in the primary lesion site [127]. This so called post-stroke secondary degeneration has been linked to neurological deficits such as depression and cognitive impairment [128, 129], and can affect motor function-related outcome [127]. However, the underlying molecular mechanisms are not clear. In murine models of Alzheimer’s disease, both neuronal and microglial C3aR signaling has been implicated in contributing to neurodegeneration [35, 50]. Given that synaptic dysfunction and loss may be the initial steps in neurodegeneration [130, 131], studies on the involvement of the complement system, C3a-C3aR and in particular C3b-CR3 signaling in the elimination of synapses in the regions affected by secondary degeneration in the post-stroke brain are warranted.

## Future Directions

Whereas complement activation, and C3a in particular, can contribute to endothelial cell activation, inflammatory cell recruitment and tissue injury in the acute phase after cerebral ischemia, C3a-C3aR signaling evidently also supports functional recovery by stimulating post-stroke neural plasticity including cell replacement, reorganization of axonal circuitry, and synaptogenesis. After intranasal administration, therapeutic peptides are transported mainly via peri-vascular bulk flow along the olfactory and trigeminal nerves and reach the brain and the cerebrospinal fluid within minutes [132]. The findings of the plasticity- and recovery-promoting effects of C3a given via this clinically highly feasible and non-invasive route are particularly intriguing as the therapeutic benefit was achieved when treatment was initiated as late as 7 days after stroke. The available data point to intranasal delivery of C3aR agonists in the post-acute phase as an attractive approach to improve functional recovery after ischemic stroke. Given the lack of functional improvement promoting pharmacological therapies in the post-acute and chronic phase after stroke, clinical translation of these findings is warranted. The broad therapeutic window would allow the majority if not all stroke survivors to benefit from such a treatment.
